# Burden of Cardiovascular Diseases in Nepal from 1990 to 2019: The Global Burden of Disease Study, 2019

**DOI:** 10.1155/2023/3700094

**Published:** 2023-06-19

**Authors:** Achyut Raj Pandey, Meghnath Dhimal, Niraj Shrestha, Dikshya Sharma, Jasmine Maskey, Raja Ram Dhungana, Bihungum Bista, Krishna Kumar Aryal

**Affiliations:** ^1^HERD International, Kathmandu, Nepal; ^2^Nepal Health Research Council, Kathmandu, Nepal; ^3^Abt Associates, Kathmandu, Nepal; ^4^Sambhav (Possible), Kathmandu, Nepal; ^5^Oxford University Clinical Research Unit, Lalitpur, Nepal; ^6^Nepal Family Development Foundation, Lalitpur, Nepal; ^7^Public Health Promotion and Development Organization, Kathmandu, Nepal

## Abstract

Cardiovascular diseases (CVDs) have emerged as the leading cause of deaths worldwide in 2019. Globally, more than three-quarters of the total deaths due to CVDs occur in low- and middle-income countries like Nepal. Although increasing number of studies is available on the prevalence of CVDs, there is limited evidence presenting a complete picture on the burden of CVDs in Nepal. In this context, this study aims to provide comprehensive picture on the burden of CVDs in the country. This study is based on the Global Burden of Disease (GBD) study 2019, which is a multinational collaborative research covering 204 countries and territories across the world. The estimations made from the study are publicly available in the GBD Compare webpage operated by the Institute for Health Metrics and Evaluation (IHME), University of Washington. This article makes use of those data available on the GBD Compare page of IHME website to present the comprehensive picture of the burden of CVDs in Nepal. Overall, in 2019, there were an estimated 1,214,607 cases, 46,501 deaths, and 1,104,474 disability-adjusted life years (DALYs) due to CVDs in Nepal. The age-standardized mortality rates for CVDs witnessed a marginal reduction from 267.60 per 100,000 population in 1990 to 245.38 per 100,000 population in 2019. The proportion of deaths and DALYs attributable to CVDs increased from 9.77% to 24.04% and from 4.82% to 11.89%, respectively, between 1990 and 2019. Even though there are relatively stable rates of age-standardized prevalence, and mortality, the proportion of deaths and DALYs attributed to CVDs have risen sharply between 1990 and 2019. Besides implementing the preventive measures, the health system also needs to prepare itself for the delivery of long-term care of patients with CVDs which could have significant implications on resources and operations.

## 1. Introduction

Deaths and disability due to cardiovascular diseases (CVDs) are largely preventable. However, CVDs remain the foremost cause of death and disability worldwide in 2019 [1, 2]. Over a third (32.84%) of the total deaths and approximately one-sixth (15.52%) of the total DALYs across the globe are attributed to CVDs [1, 3, 4].

CVDs include ischemic heart disease (IHD), stroke, intracerebral haemorrhage, rheumatic heart disease (RHD), hypertensive heart disease, peripheral arterial disease, and several other cardiac and vascular conditions. Four out of five CVD-related deaths are due to heart attacks and strokes, with almost one-third of these deaths occuring in people under 70 years of age [5]. With well-designed screening programmes and appropriate use of scarce resources, much of the suffering from CVDs [6] and the associated economic burden can be alleviated. Raised blood pressure (BP) and blood glucose, dyslipidaemia, overweight, and obesity are common risk factors of CVDs, and almost all these risk factors can be managed in primary care facilities.

In recent decades, the burden of CVDs has been rising in almost all parts of the world [7], and this has particularly affected low- and middle-income countries (LMICs) [8]. Although the risks associated with CVDs are easily identifiable, more than three-quarters of the total deaths due to CVDs globally occur in LMICs. Moreover, about 40% of these deaths are premature [9]. Besides the lack of appropriate screening programmes, the high burden of CVDs could be related to ageing population resulting from increased life expectancy [10] and higher prevalence of risk factors such as smoking, physical inactivity, obesity, diabetes, dyslipidaemia, and hypertension [2, 11–14]. Evidence from the Prospective Urban and Rural Epidemiology (PURE) study, a cohort study covering diverse populations from 21 high-income, middle-income, and low-income countries, suggests that air pollution increases the risk of CVDs, particularly stroke [15]. LMICs often have a limited capacity to diagnose CVDs at an early stage and provide treatment, leading to complications and often leading to premature deaths [2]. Adults living in LMICs like Nepal are at high risk of death, disability, and financial burden resulting from CVDs [2]. At a macroeconomic level, the increasing burden of CVDs leads to a substantial economic burden because of long-term chronic care [5].

Without cost-effective interventions to strategically address the rising burden of CVDs, it will be a challenging task to achieve sustainable development goal 3, which includes reduction in premature deaths from noncommunicable diseases (NCDs) by one-thirds by 2030 [16]. Previous study suggests that the countries are not on track to achieve this goal because of the slow decline in deaths from CVDs [2]. Estimating the burden of CVDs is essential for programme and policy design. Past studies have also indicated that CVD incidence and mortality rates have been continuously increasing since 1990, with the burden greater amongst males and older age groups [17]. Similarly, the population-based prevalence of coronary artery disease in the 20 years and above age group in Nepal shows a prevalence of 2.90% [18].

Nepal lacks data on the trend of specific CVDs such as ischemic heart disease, stroke rheumatic heart disease, hypertensive heart disease, atrial fibrillation and flutter, aortic aneurysm, nonrheumatic valvular heard disease, cardiomyopathy and myocarditis, endocarditis, peripheral artery disease, and other cardiovascular and circulatory diseases disaggregated by the age group and sex. This study aims to present the burden of CVDs in Nepal using the latest GBD 2019 data.

## 2. Methods

This study used the publicly accessible data (2019 edition) available in the GBD Compare page housed within the Institute for Health Metrics and Evaluation (IHME) website [1]. GBD 2019 is a multinational collaborative research study that estimated the disease burden in 204 countries and territories between 1990 and 2019 [7, 19]. The study uses various data sources such as censuses, household surveys, health service use, civil registration and vital statistics, disease registries, air pollution monitors, satellite imaging, disease notifications, and other sources between 1990 and 2019 in making estimates of the burden of disease (BoD) [4].

The most common measures that the GBD study use to estimate the the Burden of Disease (BoD) are disability-adjusted life years (DALYs) and mortality (death). DALYs combines years of life lost (YLLs) and years lived with disability (YLDs). YLLs from different diseases are calculated based on data from vital registration with medical certification (or cause of death assessed using verbal autopsies where medical certification of the causes of death is not available). YLDs are calculated using outpatient and inpatient data from health facilities, disease registries, and direct measurements in some cases. The sum total of YLLs and YLDs provides DALYs. Using data from censuses, vital registration systems, and periodic and standalone household surveys, GBD researchers estimate adult and child mortality. The number of premature death and disability attributable to different risk factors are calculated using data on exposure, impact, and risk factors [4, 20]. A total of 86,249 data sources were used in GBD 2019 [4], including 389 data sources from Nepal [20, 21].

In GBD 2019, to present the BoD of different diseases, 369 diseases and injuries were organised into a levelled cause hierarchy from the three broadest causes of death and disability at level 1 to the most specific causes at level 4 [4, 22]. Level 1 causes include aggregates of NCDs, injuries, and a broad category combining communicable diseases, maternal and neonatal, and nutritional diseases (CMNN diseases). At level 2, there are 22 disease and injury aggregate groupings such as CVDs, respiratory infections, tuberculosis (TB), and transport injuries. Level 3 includes specific causes such as stroke, hypertensive and rheumatic heart disease, and peripheral artery disease. Likewise, level 4 includes further detailed classification including alcoholic cardiomyopathy and myocarditis [4, 20, 22].

For this article, we extracted data from level 2 which includes data on prevalence, mortality, and DALYs attributable to CVDs. Data for specific CVDs such as IHD, RHD, hypertensive heart disease, atrial fibrillation and flutter, nonrheumatic valvular heart disease, cardiomyopathy and myocarditis, endocarditis, peripheral artery disease, stroke and other cardiovascular, and circulatory diseases were extracted from level 3 [7]. Both age-standardized and age-unstandardized rates have been reported. Age-standardization was performed via the direct method, applying a global age structure from the year 2019. GBD 2019 uses the standard case definitions for the different causes of death covered in the study [7]. Uncertainty intervals (UIs) were produced by selecting the 25th and 975th values out of 1000 ordered draws from the posterior distribution [4]. The detailed definitions of the causes of death used in this study are presented in the previous publications [7].

## 3. Results

There were an estimated 1,214,607 prevalent cases of CVDs in both sexes in 2019. About half (52.83%) of these cases were male (not shown in table) in Nepal. The all-age prevalence rate increased from 2,783.24 cases per 100,000 population (95% UI: 2,604.71–2,964.44) in 1990 to 3,993.27 cases per 100,000 population (95% UI: 3,740.42–4,236.51) in 2019. Among males, the prevalence rate increased from 3,065.62 per 100,000 population (95% UI: 2,866.08–3,268.48) to 4,426.36 per 100,000 population (95% UI: 4,153.15–4,690.52) between 1990 and 2019. Similarly, among females, the prevalence rate increased from 2499.41 (95% UI: 4638.57–5253.07) to 3598.83 (95% UI: 4,431.64–5,013.95) per 100,000 population between 1990 and 2019. In the same period, the age-standardized prevalence rate decreased from 5,553.69 (95% UI: 5,228.51–5,872.43) to 5,341.91 (95% UI: 5,038.18–5,647.1) in both sexes, 6,138.23 (95% UI: 5,776.32–6,492.63) to 6,015.68 (95% UI: 5,671.84–6,351.62) in males, and 4,940.13 (95% UI: 4,638.57–5,253.07) to 4,720.9 (4,431.64–5,013.95) in females (Table 1).

**Figure 1:**
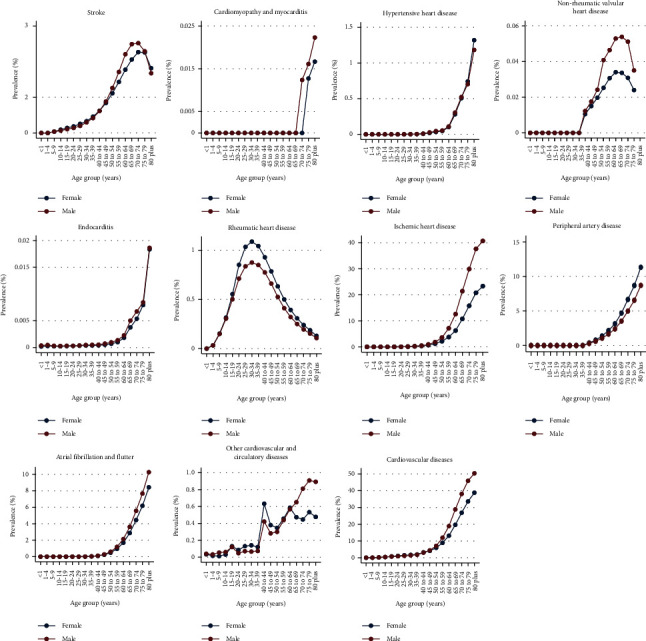
Prevalence of different CVDs (per 100,000 population) among different age groups of the male and female population.

The prevalence rate of CVDs increased steadily with age, reaching a peak at the 80 plus age group. In 80+ population, the prevalence is approximately 44% in both sexes; 50% in males and 38% in females. A small proportion of the total prevalent cases are contributed by the population below 20 years of age. In 2019, the most common CVDs were IHD and peripheral artery disease (Figure 1).

**Figure 2:**
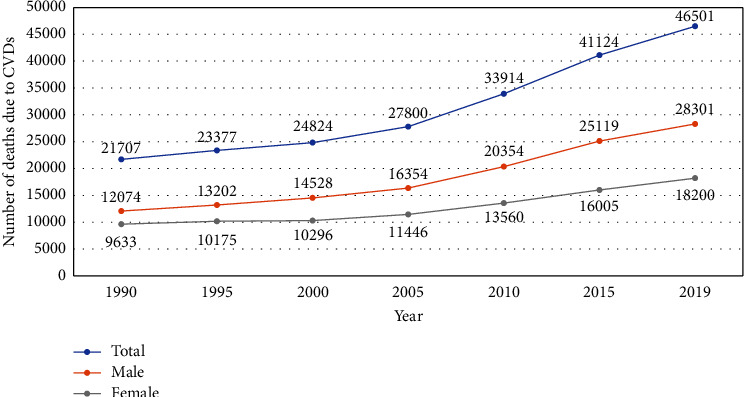
Deaths due to CVDs from 1990 to 2019 in Nepal.

A total of 46,501 CVD deaths were estimated to have occurred in 2019, with 28,301 deaths among males and 18,200 deaths among females (Figure 2).

**Figure 3:**
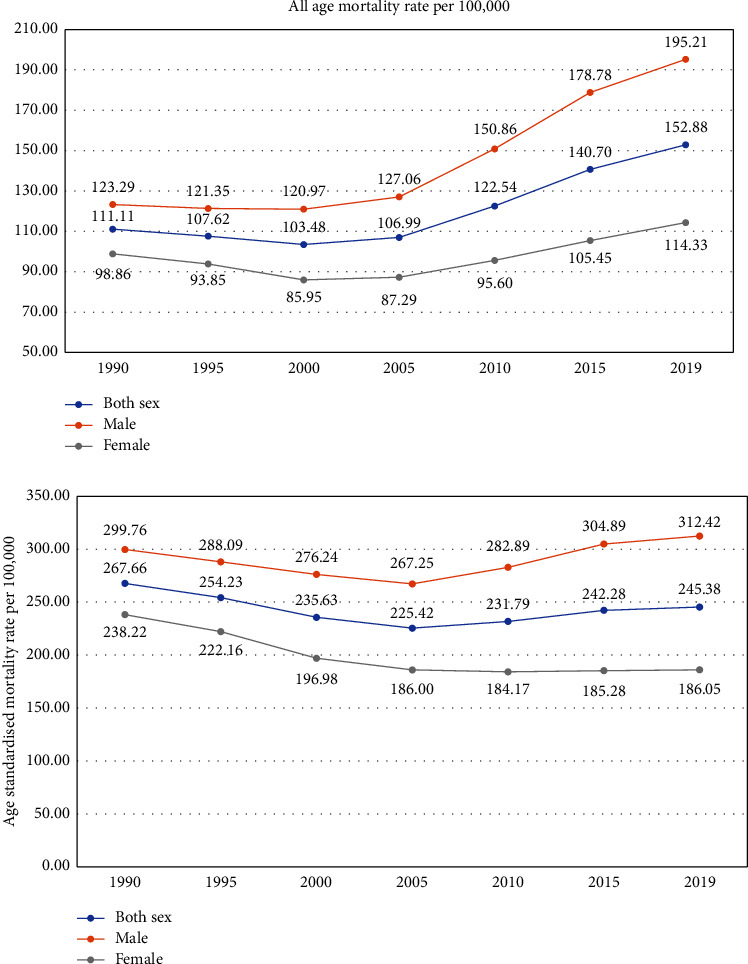
All age and age-standardized mortality rate due to CVDs per 100,000 population.

The all-age mortality rate for CVDs increased from 111.11 deaths per 100,000 population in 1990 to 152.88 deaths per 100,000 population in 2019. However, the age-standardized mortality rate for CVDs reduced marginally from 267.60 per 100,000 population to 245.38 per 100,000 population in 2019. The increase in mortality rate among males is relatively abrupt compared to females. The all-age mortality rate for CVDs increased from 123.29 deaths per 100,000 population in 1990 to 195.21 deaths per 100,000 population in 2019 among males. Unlike the age-standardized mortality rates in both sexes, the age-standardized mortality rate for CVDs has increased from 299.76 per 100,000 population to 312.42 per 100,000 population between 1990 and 2019. Among females, the all-age mortality rate increased from 98.96 per 100,000 population to 114.33 per 100,000 population between 1990 and 2019, while the age-standardized mortality rate decreased from 238.22 per 100,000 population to 186.05 per 100,000 population in the same period (Figure 3).

There has been a steep rise in the proportion of deaths attributable to CVDs out of total deaths. In 1990, approximately 9.77% (95% UI: 8.41–11.81) of the total deaths were due to CVDs, which has increased to 24.04% (95% UI: 21.73–26.75) of the total deaths in 2019. In 1990, 10.37% (95% UI: 8.80–12.81) of the deaths in males and 9.11% (95% UI: 7.2–11.48) of the deaths in females were due to CVDs, which increased to 26.79% (95% UI: 23.9–31.13) of the total deaths in males and 20.73% (95% UI: 17.33–24.11) of the total deaths in females in 2019 (Table 2).

In infants below one year of age, 0.12% (95% UI: 0.07–0.18) of the deaths are due to CVDs in 2019, which increased steadily with age. The highest proportion of deaths being attributable to CVDs in the age group 65–69 years, 30.44% (95% UI: 26.03–35.39). The mortality rate due to CVDs is higher in males than in females (Supplementary file 1). In 2019, approximately 0.46% (214 deaths) of the total deaths from CVDs is from the age groups below 20 years. Ages below 20 years contributed 0.52% (147 deaths) of the total CVD deaths among males and 0.37% (67 deaths) among females in 2019 (not shown in the table).

Among CVDs, in 2019, IHD had the highest mortality rate and had the largest share in total deaths that occurred in Nepal. The all-age mortality rate for IHD was 78.06 (95% UI: 61.53–94.72) per 100,000 population and the age-standardized mortality rate was 123.99 (95% UI: 98.62–148.27) per 100,000 population in both sexes which accounted approximately 12.27% (95% UI: 10.69–14.09) of the total deaths in 2019. IHD had the all-age mortality rate of 109.38 (95% UI: 83.58–134.52) per 100,000 population and an age-standardized mortality rate of 173.68 (95% UI: 133.43–213.06) per 100,000 population in males which accounted 15.01% (95% UI: 12.92–17.62) of the total deaths in 2019. The second leading cause of deaths among CVDs was stroke with the all-age mortality rate of 49.94 (95% UI: 39.33–61.4) per 100,000 population and age-standardized mortality rate of 80.44 (95% UI: 63.44–98.61) per 100,000 population in 2019 (Table 3).

**Figure 4:**
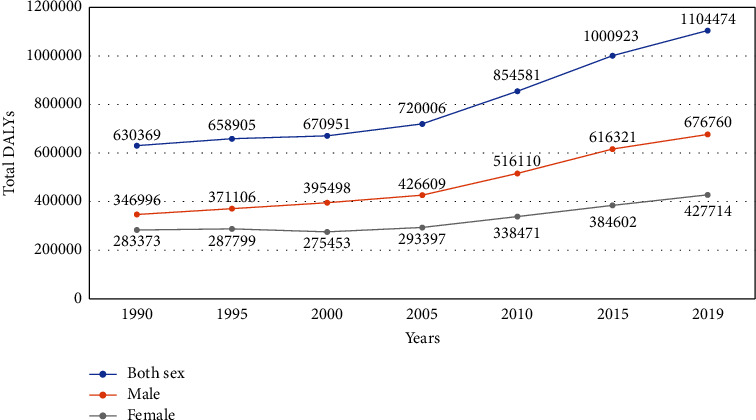
DALYs due to CVDs from 1990 to 2019.

A total of and 1,104,474 DALYs were attributed to CVDs in 2019, 6,76,760 DALYs among male and 4,27,714 DALYs among females (Figure 4).

In 1990, 3,226.61 (2,663.46–3,970.16) DALYs per 100,000 were due to CVDs (4.07% of the total DALYs, 95% UI: 3.45–4.87) which increased to 3,631.18 (2,926.9–4,340.92) DALYs per 100,000 (11.89% of the total DALYs, 95% UI: 10.14–13.82). In males, 4,668.02 (3,666.2–5,645.85) DALYs per 100,000 (14.28% of the total DALYs, 11.95–16.95) were due to CVDs in 2019, which is an increase from 3,543.25 (95% UI: 2,895–4,428.79) per 100,000 (4.38%, 95% UI: 3.64–5.34) in 1990. In females, 2,686.89 (95% UI: 2,129.38–3,297.54) DALYs per 100,000 (9.41% of total DALYs, 95% UI: 7.65–11.31) were due to CVDs in 2019, which is an increase from 2,908.36 (95% UI: 2,298.39–3,667.29) per 100,000 (3.74%, 95% UI: 3.02–4.64) in 1990. Despite the increase in the age-standardized prevalence, the age-standardized DALYs decreased from 6,099.2 (95% UI: 5,050.45–7,507.92) per 100,000 in 1990 to 4,962.14 (95% UI: 4,021.22–5,854.19) per 100,000 in 2019. However, in males, the age-standardized DALYs has remained stable; 6,671.06 (95% UI: 5,483.6–8,334.97) DALYs per 100,000 in 1990 and 6,379.73 (95% UI: 5,049.35–7,676.34) DALYs per 100,000 in 2019. Unlike males, the age-standardized DALYs decreased from 5,525.86 (95% UI: 4,284.74–7,001.57) in 1990 to 3,687.93 (95% UI: 2,940.81–4,488.52) in females in 2019. The decline in age-standardized DALYs seems to be largely due to the decline in age-standardized DALYs for CVDs among females while it has remained relatively stable among males (Table 4).

Like the prevalence and mortality rate, the proportion of DALYs due to CVDs increased steadily with the age (Supplementary file 2).

Among CVDs, IHD was the leading cause of DALYs and was responsible for 5.97% (95% UI: 4.9–7.15) of the total DALYs in both sexes combined, 7.93% (95% UI: 6.38–9.57) of the total DALYs in male and 3.93% (95% UI: 2.88–5.08) of the total DALYs in female in 2019. Similarly, 3.74% (95% UI: 3.08–4.57) of the total DALYs in both sexes combined, 4.21% (95% UI: 3.41–5.32) of the total DALYs in male and 3.24% (95% UI: 2.51–4.21) of the total DALYs in female, were due to stroke in 2019. About more than half of the total DALYs due to CVDs in 2019 were due to IHD; 2,490.53 (95% UI: 1,961.78–3,040.38) DALYs per 100,000 among both sexes, 3,543.47 (95% UI: 2,716.66–4,396.01) DALYs per 100,000 among male and 1,548.03 (95% UI: 1,118.11–2,018.9) DALYs per 100,000 among female (Table 5).

## 4. Discussion

In Nepal, the all-age prevalence rate of CVDs has increased from 2,783.24 per 100,000 population in 1990 to 3,993.27 per 100,000 population in 2019. During the same period, the age-standardized prevalence rate decreased from 5,553.69 to 5341.91 per 100,000 population. Globally, while the all-age prevalence rate of CVDs increased by 33% from 5,069 cases per 100,000 to 6,762 cases per 100,00 between 1990 and 2019, the age-standardized prevalence rate decreased by 4% from 6728 to 6,432 cases per 100,000 [1]. Similarly, in South Asia, the all-age prevalence rate of CVDs increased by 50% from 3304 cases per 100,000 to 4,944 cases per 100,000 between 1990 and 2019, and the age-standardized prevalence rate increased marginally by 3% from 6,050 to 6,219 cases per 100,000 [1]. Increase in the all-age prevalence rate with a decline or marginal increment (in the case of South Asia) in the age-standardized prevalence rate of CVDs indicates that the changes in prevalence rates are largely influenced by the change in the population structure due to population ageing as has been witnessed in the past few decades with increasing life expectancy [1]. In the same period, the all-age mortality rate from CVDs increased by 6% globally and by 30% in South Asia. The all-age mortality rate from CVDs increased by 32% in India, 70% in Bhutan, and 61% in Bangladesh. The age-standardized mortality rate from CVDs from 1990 to 2019 decreased by 32% globally and 20% in South Asia. Among different countries in South Asia, the age-standardized mortality rate declined by 23% in India, 12% in Bhutan, and 15% in Bangladesh [1].

While the age-standardized prevalence and mortality rates in Nepal have declined marginally from 1990 to 2019, the proportion of deaths attributable to CVDs increased from 9.77% to 24.04% in both sexes (10.37%–26.79% in males and 9.11%–20.73% in female). Similar trend has been observed in neighbouring countries. The age-standardized mortality rate has shown a declining trend, despite a relatively stable slight increase in all-age mortality rates globally [1, 7]. In India, the age-standardized mortality rate declined from 332.83 deaths per 100,000 to 356.37 deaths per 100,000, while the proportion of deaths attributable to CVDs has increased from 14.52% to 27.4% of the total deaths from 1990 to 2019 [1]. Similar pattern has also been noted in other neighbouring countries including Bhutan and Pakistan [1]. This could be because of the successes in reducing mortality due to other health conditions than CVDs, leading to dramatic increase in the share of deaths attributable to CVDs while age-standardized mortality decreased marginally or is relatively stable.

In 2019, IHD (the all-age prevalence rate of 2,079.24 and the age-standardized prevalence rate of 2,991.92 cases per 100,000 population) and peripheral artery disease (the all-age prevalence rate of 615.20 and the age-standardized prevalence rate of 898.99 cases per 100,000 population) were the two most prevalent CVDs in Nepal. Similarly, 12.27% of the total deaths and 5.97% of the total DALYs were due to IHD in 2019. IHD and peripheral artery disease were also the two most prevalent CVDs worldwide. Globally, 16.17% of the total deaths and 7.19% of the total DALYs were due to IHD in 2019. Among neighbouring countries, IHD and peripheral artery disease were the two most prevalent conditions in India, Bhutan, Maldives, and Sri Lanka, and stroke appeared to be the second most prevalent condition in other countries such as Pakistan and Bangladesh. The proportion of total deaths attributable to IHD was 16.17% in India, 15.62% in Bhutan, 17.67% in Sri Lanka, 12.22% in Pakistan, and 15.41% in Bangladesh [1]. The proportion of total DALYs attributable to IHD was 7.97% in India, 6.86% in Bhutan, 8.54% in Sri Lanka, 5.47% in Pakistan, and 7.13% in Bangladesh in 2019 [1]. Findings from Nepal seem to be comparable with other neighbouring countries.

Often referred to as “causes of the causes,” the underlying causes that have led to the increase in prevalence, mortality, and burden of CVDs are the social, economic, and cultural changes resulting from globalisation and urbanisation [5]. The behavioural risk factors for CVDs such as tobacco use, unhealthy diet, low level of physical activity, and harmful use of alcohol gradually show up in the form of intermediate risk factors such as raised BP, raised blood glucose, dyslipidaemia, and high body mass index (BMI) [1, 5]. Effectively addressing these risk factors can also offer an advantage in controlling diseases other than CVDs, such as diabetes, kidney disease, and cancer. Previous studies in Nepal indicate that risk factors for CVDs often show clustering [23], thereby demanding comprehensive interventions addressing multiple risk factors simultaneously. Preventive strategies for CVDs could promote a healthy diet, adequate levels of physical activity, and reduction in tobacco and alcohol use. Simultaneously, efforts should be made to create a conducive environment where lifestyle modification can be achieved and sustained.

Apart from behavioural risk factors, environmental risk factors like particulate matter pollution, including ambient and indoor air pollution, increases the risk of CVDs [1, 15, 24]. Broader, interdisciplinary teams working collaboratively to promote a healthy environment with community engagement can be effective [11]. The current federal structure of Nepal, where local level governments are responsible for basic health services [23] and also oversee multiple sectors could offer an opportunity for multisectoral interventions to control CVDs. Likewise, locally contextualized health promotional interventions could prove effective for addressing behavioural risk factors. Apart from creating conducive environments for promoting physical activity levels through the construction of public parks, cycle lanes, physical fitness centres, yoga, and meditation centres, local governments could work further in reducing indoor air pollution and ambient air pollution, which could further reduce the prevalence, mortality, and loss of DALYs due to CVDs. Shifting high polluting industries from residential areas to other locations and controlling and regulating vehicular emissions could be a useful strategy to reduce ambient air pollution, while promoting clean fuels in domestic use could help reduce indoor air pollution.

The WHO recommends the HEARTS strategy: healthy lifestyle counselling, evidence-based treatment protocols, access to essential medicines and technology, risk-based CVD management, team-based care, and a system for monitoring to effectively address the burden of cardiovascular health in any countries [25].

Healthy lifestyle counselling could cover risk factors of CVDs such as unhealthy diet, tobacco use, insufficient physical activity, and harmful use of alcohol as important contributors to CVDs [25]. As suggested by the WHO, the “theory of 5As” (asking patients about risk factors, advising them in a simple, clear, and personalised manner, assessing the patients readiness to change, assisting them to develop a plan, and arranging follow-ups contacts by phone and in person) could be useful [25]. Apart from counselling and assisting patients in behaviour change, interventions at the national level intending to discourage unhealthy behaviours could reduce the consumption of salted products and sweetened beverages. Taxation on sugar-sweetened beverages was found to be a cost-effective intervention in Mexico [26]. Apart from taxation, foods labelled as high in salt has been found to reduce the consumption of such products [9, 27].

Systematic review by Gheorghe et al. on the economic burden of CVDs and hypertension reports that the annual CVDs care costs exceed health expenditure per capita in most LMICs. Generally, NCDs, including CVDs, require long-term care [28]. Prolonged care requires effective mobilisation of the fund within the health system. Hence, to ensure that people receive health care services without financial hardships, efforts should be made to strengthen the prepaid contributions, risk pooling, and strategic purchasing of health systems [29].

The country should also promote healthy dietary habits such as increased intake of whole grains, legumes, fruits and vegetables, fibre-rich foods, and food rich in polyunsaturated fatty acids and reduced intake of sweetened beverages, salted foods, high sodium foods, trans fat, and red meat. Health education messages are not sufficient for the promotion of a healthy diet and depend on multiple other factors such as the cost of processed food versus fresh produce, distance to the fruit and vegetable markets, and the cost of fruits and vegetables [5, 30, 31]. A functioning dietary guideline can be effective in promoting a healthy diet. One of the previous studies concluded that Nepal's dietary policies are not aligned with the country's disease situation [32]. Per-capita energy consumption and sugar and sweeteners consumption have witnessed a significant rise in the past few decades [33], which could be the result of ineffective policy initiatives to promote a healthy diet to address the increased burden of NCDs including CVDs.

Promoting physical activity could also be an effective strategy to prevent cardiovascular disease incidence and associated mortality. UK Biobank study revealed that high levels of cardiorespiratory fitness could reduce the risk of coronary heart disease by 49% and the risk of atrial fibrillation by 60% among individuals with a high genetic risk for these diseases [34]. Rotterdam study (15 years follow-up study among adults aged 55 years) revealed that overweight and obese individuals with high physical activity levels did not have an increased risk of normal weight counterparts, while overweight and obese individuals with low physical activity levels were found to be at an increased risk [35]. Motivational strategies such as the setting up of exercise rooms in enterprises, having higher than a certain number of staffs, encouraging cycling, setting up specific cycle lanes in cities, setting up specific physical activity goals, and encouraging self-monitoring tools such as pedometers or accelerometers could be useful. Similarly, setting up park greenery in different locations where individuals can do jogging or exercise can also be useful as people often find difficulties in city areas to find peaceful and less polluted areas for physical activity, morning walks, and jogging, among others.

Tobacco consumption is the single most preventable cause of CVDs. The previous studies in European countries indicate that smoking almost doubles the 10-year CVDs mortality rate [36, 37]. In the United States of America, a little under a third of (30%) of CVD deaths were attributable to smoking [37, 38]. Countries worldwide face a high burden of tobacco consumption irrespective of their economic status and advancement in the health sector. However, the impacts remain more pronounced in LMICs, which bear the largest share of deaths from CVDs globally. Strategies to reduce the consumption of tobacco products could be crucial to reducing the burden of CVDs [39]. For instance, in Nepal, approximately 44.8% of the current smokers had thought of quitting because of warning labels on cigarette packages and approximately 19.4% tried to quit smoking, which indicates that warning labels on cigarettes could effectively reduce the smoking prevalence [40]. Increasing tax on tobacco products, on the other side, discourages people from consuming such products and generates additional resources for the health system [41, 42].

In our study, the prevalence rate of CVDs was 44190.77 per 100,000 population in the 80 years and above age group. Besides population-based prevention and promotion-related services, the health system also needs to prepare itself to provide treatment services to an increasing number of people with CVDs. As the evidence on the outcome of different treatment options are continuously updated, the adoption of evidence-based treatment protocols is essential [43], and advocacy for this among policy makers at the national and provincial levels and programme managers at different levels of the health care system is also essential.

Early diagnosis and treatment of intermediate risk factors such as raised BP, raised blood glucose levels, and dyslipidaemia could prevent the progression to CVDs and, thus, prevent the associated complications and deaths. As a notable proportion of these intermediate risk factors go undetected at the community level, screening programmes at primary level health facilities could be useful. Opportunistic screening programmes that involve screening of high-risk populations for cardiovascular risk factors while they visit health facilities for other diseases can also be useful in the early detection of CVDs and its risk. Previous studies have revealed that such screening programmes at the primary health care level could be effective in the early detection of the disease [44, 45]. Furthermore, the extensive network of female community health volunteers (FCHVs) across the country could offer an opportunity for the screening of risk factors such as raised BP and high BMI among high-risk population at the community level. One of the previous studies in Nepal also indicates that such a strategy could be cost-effective and can be an option to reduce the prevalence of undiagnosed raised BP at the community level [46]. FCHVs could also refer high-risk populations to health facilities for regular monitoring of plasma glucose and dyslipidaemia. Local governments could also implement a campaign-like screening programme for screening CVD risk factors such as raised BP, high BMI, dyslipidaemia, and raised plasma glucose at the community level.

As part of the package of essential NCDs (PEN), the Government of Nepal has envisioned making services such as the detection of hypertension, diabetes, and CVD risk assessment available at the health post level [47, 48]. Raised BP, one of the main risk factors for CVDs, can be treated with simple and effective yet affordable interventions at the primary health care level. Despite the ease with which raised BP can be diagnosed and treated, it is largely overlooked as hypertensives may not present symptoms. Undiagnosed and untreated hypertension could pose a serious risk of CVDs and are often referred to as “silent killer” [49]. Meanwhile, the readiness of the health service provider is equally important.

Different levels of government should also need to ensure access to essential medicines and technology. An earlier study that analysed data from a health facility survey (940 health facilities throughout the country) revealed that health facilities had very limited readiness to respond to CVDs, including the availability of guidelines (for diagnosis and treatment) and staff training on CVD treatment [50]. Health facilities were also found to have very limited readiness in terms of availability of equipment and essential medicines. Hence, it would be essential to strengthen service delivery in addition to rolling out the required health service package.

With the increasing burden of NCDs, including CVDs, the country should also focus on developing a sustainable financing mechanism and a self-sufficient health system. Nepal is in the process of graduating from the list of least developed countries and may soon outgrow from the development assistance in health (DAH) with economic progress. The country needs to prepare itself for moving forward from dependence on DAH and out-of-pocket expenditure to a more robust government and prepaid health care spending system.

## 5. Strengths and Limitations

Advanced statistical techniques have been applied in the BoD 2019 study, ensuring comparability of the health data recorded using the International Classification of Diseases system across the countries and territories considered in the study. This improves the comparability of CVDs burden in Nepal with other countries. Nepal lacks the monitoring system for determining the causes of death nor are there any recognized types of verbal or social autopsy to document CVDs. In this context, GBD data, computed through robust statistical techniques, can be used to set public health priorities and inform policymaking when it comes to CVDs, as reliable health statistics on the condition are scarce [17].

Owing to difficulties in obtaining death certificate and problem in identifying an underlying cause of deaths, mortality- and burden-related data may have been slightly underestimated or overestimated. The availability of diagnostic technologies for CVDs in countries such as Nepal may have influenced the estimate of specific diseases, since restricted access may lead to lower reported prevalence rates [7].

## 6. Conclusion

CVDs have emerged as a major public health problem in Nepal, contributing a large and increasing share of deaths and DALYs in 2019. One major reason could be the progress achieved in reducing mortality and DALYs from other diseases, while these have remained stable for CVDs. Others could be changing lifestyles, particularly diet and physical activity. Addressing the increasing burden of CVDs in Nepal requires addressing the risk factors for these diseases at multiple fronts with the collaboration of different sectors. Opportunistic screening at the health facility level and community screening programmes led by FCHVs for risk factors of CVDs such as raised BP, high BMI, raised cholesterol, and raised plasma glucose could be useful in the early detection and prevention of disease. The health system also needs to prepare itself for the delivery of long-term care of patients with CVDs that could have significant implications for the resources and current organization of the health system.

## Figures and Tables

**Table 1:** Prevalence rate (per 100,000 population) of CVDs from 1990 to 2019.

Years	Both sex		Male		Female	
	All-age prevalence rates (95% UI)	Age-standardized prevalence rates (95% UI)	All-age prevalence rates (95% UI)	Age-standardized prevalence rates (95% UI)	All-age prevalence rates (95% UI)	Age-standardized prevalence rates (95% UI)
1990	2,783.24 (2,604.71–2,964.44)	5,553.69 (5,228.51–5,872.43)	3,065.62 (2,866.08–3,268.48)	6,138.23 (5,776.32–6,492.63)	2,499.41 (2,327.82–2,687.89)	4,940.13 (4,638.57–5,253.07)
1995	2,839.86 (2,674.15–3,007.68)	5,523.79 (5,238.38–5,810.5)	3,123.65 (2,952.96–3,303.37)	6,120 (5,824.27–6420.52)	2,555.06 (2381.13–2,734.89)	4,906.21 (4,622.81–5,198.81)
2000	2,988.28 (2,806.02–3,182.86)	5,556.57 (5,256.81–5,864.2)	3,298.06 (3,102.24–3,502)	6,183.81 (5,839.59–6,522.98)	2,677.7 (2,489.77–2,882.47)	4,913.84 (4,612.1–5,236.02)
2005	3,241.18 (3,058.55–3,431.72)	5,571.66 (5,285.69–5,855.29)	3,606.02 (3,405.72–3,808.01)	6,220.5 (5,913.72–6,521.02)	2,883.07 (2,697.85–3,096.34)	4,911.66 (4,638.48–5,205.61)
2010	3,517.4 (3,293.75–3,747.82)	5,502.57 (5,190.5–5,823.02)	3,900.93 (3,668.08–4,148.75)	6,119.67 (5,771.43–6,484.39)	3,152.62 (2,930.9–3,395.28)	4,886.91 (4,567.89–5,207.13)
2015	3,752.25 (3,539.25–3,963.24)	5,374.67 (5,093.13–5,642.37)	4,183.47 (3,949.28–4,414.15)	6,035.5 (5,718.52–6,333.61)	3,353.06 (3,131.5–3,589.07)	4,741.94 (4,470.34–5,026.36)
2019	3,993.27 (3,740.42–4,236.51)	5,341.91 (5,038.18–5,647.1)	4,426.36 (4,153.15–4,690.52)	6,015.68 (5,671.84–6,351.62)	3,598.83 (3,352.2–3,844.89)	4,720.9 (4,431.64–5,013.95)

**Table 2:** Proportion of total deaths attributable to CVDs.

Years	Proportions of deaths in both sex (95% UI)	Proportions of deaths in male (95% UI)	Proportions of deaths in female (95% UI)
1990	9.77 (8.41–11.81)	10.37 (8.80–12.81)	9.11 (7.2–11.48)
1995	11.60 (10.2013.65)	12.38 (10.80–14.87)	10.73 (8.85–13.07)
2000	13.91 (12.48–16.04)	15.04 (13.41–17.78)	12.58 (10.73–15.01)
2005	16.52 (14.59–19.15)	17.86 (15.55–21.83)	14.91 (12.96–17.36)
2010	19.67 (17.54–22.67)	21.58 (18.85–26.15)	17.37 (15.33–19.71)
2015	21.29 (19.15–23.92)	23.55 (20.95–27.72)	18.51 (15.81–21.02)
2019	24.04 (21.73–26.75)	26.79 (23.9–31.13)	20.73 (17.33–24.11)

**Table 3:** Mortality rates (per 100,000 population) and proportion of deaths attributable to different CVDs in 2019.

	Mortality rates of both sex		% of total deaths in both sex (95% UI)	Mortality rates in male		% of total deaths in male (95% UI)	Mortality rates in female		% of total deaths in female (95% UI)
	All-age mortality rates (95% UI)	Age-standardized mortality rate(95% UI)		All-age mortality rates (95% UI)	Age-standardized mortality rates(95% UI)		All-age mortality rates (95% UI)	Age-standardized mortality rates(95% UI)	
Ischemic heart disease	78.06 (61.53–94.72)	123.99 (98.62–148.27)	12.27 (10.69–14.09)	109.38 (83.58–134.52)	173.68 (133.43–213.06)	15.01 (12.92–17.62)	49.53 (35.67–64.28)	80.14 (57.24–104.75)	8.98 (6.59–11.33)
Stroke	49.94 (39.33–61.4)	80.44 (63.44–98.61)	7.85 (6.72–9.3)	60.31 (46.13–77.31)	96.97 (74.69–123.26)	8.28 (6.97–10.19)	40.49 (30.35–53.15)	65.79 (49.13–85.73)	7.34 (5.72–9.28)
Rheumatic heart disease	9.23 (5.93–13.86)	13.36 (8.76–20.49)	1.45 (0.99–2.13)	9.76 (5.65–19.51)	14.41 (8.22–29.43)	1.34 (0.81–2.6)	8.75 (5.17–13.84)	12.43 (7.36–19.34)	1.59 (0.99–2.43)
Hypertensive heart disease	8.01 (5.78–11.17)	14.27 (10.41–20.24)	1.26 (0.94–1.74)	7.03 (4.53–10.23)	12.29 (8.11–17.68)	0.97 (0.66–1.36)	8.9 (5.17–14.03)	15.93 (9.38–25.05)	1.61 (0.98–2.52)
Other cardiovascular and circulatory diseases	2.88 (1.98–4.3)	4.57 (3.14–6.88)	0.45 (0.34–0.66)	3.19 (2.04–5.37)	5.09 (3.26–8.69)	0.44 (0.31–0.72)	2.59 (1.81–3.7)	4.12 (2.87–6.01)	0.47 (0.34–0.67)
Atrial fibrillation and flutter	1.87 (1.24–2.49)	4.08 (2.67–5.52)	0.29 (0.2–0.39)	1.84 (1.06–2.72)	4.13 (2.38–6.15)	0.25 (0.15–0.37)	1.9 (1.15–2.71)	4.04 (2.41–5.77)	0.34 (0.21–0.49)
Aortic aneurysm	1.07 (0.68–1.47)	1.72 (1.1–2.32)	0.17 (0.11–0.22)	1.49 (0.87–2.25)	2.37 (1.37–3.52)	0.21 (0.13–0.3)	0.68 (0.45–0.95)	1.13 (0.75–1.57)	0.12 (0.08–0.17)
Nonrheumatic valvular heart disease	0.81 (0.58–1.09)	1.35 (0.98–1.86)	0.13 (0.1–0.17)	0.87 (0.54–1.24)	1.44 (0.87–2.1)	0.12 (0.08–0.17)	0.75 (0.47–1.14)	1.27 (0.79–1.94)	0.14 (0.09–0.21)
Cardiomyopathy and myocarditis	0.44 (0.29–0.65)	0.67 (0.44–0.97)	0.07 (0.05–0.1)	0.61 (0.38–0.91)	0.92 (0.58–1.39)	0.08 (0.06–0.12)	0.28 (0.16–0.51)	0.44 (0.24–0.79)	0.05 (0.03–0.09)
Endocarditis	0.42 (0.31–0.56)	0.62 (0.45–0.82)	0.07 (0.05–0.08)	0.5 (0.32–0.71)	0.73 (0.46–1.03)	0.07 (0.05–0.09)	0.35 (0.22–0.52)	0.53 (0.33–0.79)	0.06 (0.04–0.09)
Peripheral artery disease	0.16 (0.1–0.22)	0.31 (0.19–0.43)	0.03 (0.02–0.03)	0.22 (0.11–0.33)	0.41 (0.21–0.63)	0.03 (0.02–0.04)	0.11 (0.06–0.18)	0.22 (0.12–0.35)	0.02 (0.01–0.03)

**Table 4:** DALYs per 100,000 and the proportion of DALYs attributable to CVDs.

	Both sex			Male			Female		
	DALYs per 100,000 (95% UI)		% of total DALYs (95% UI)	DALYs per 100,000 (95% UI)		% of total DALYs (95% UI)	DALYs per 100,000 (95% UI)		% of total DALYs (95% UI)
	All age	Age standardized		All age	Age standardized		All age	Age standardized	
1990	3,226.61 (2,663.46–3,970.16)	6,099.2 (5,050.45–7,507.92)	4.07 (3.45–4.87)	3,543.25 (2,895–4,428.79)	6,671.06 (5,483.6–8,334.97)	4.38 (3.64–5.34)	2,908.36 (2,298.39–3,667.29)	5,525.86 (4,284.74–7,001.57)	3.74 (3.02–4.64)
1995	3,033.49 (2,564.23–3,623.73)	5,687.27 (4,818.84–6,810.35)	4.82 (4.17–5.63)	3,410.96 (2,835.93–4,158.43)	6,357.28 (5,342.92–7,820.39)	5.27 (4.49–6.34)	2,654.68 (2,199.26–3,203.82)	5,014.06 (4,110.34–6,134.81)	4.34 (3.63–5.23)
2000	2,796.96 (2,401.46–3,260.32)	5,124.2 (4,456.37–5,954.93)	5.73 (5.07–6.55)	3,293.12 (2,779.41–3,912.57)	5,977.99 (5,117.68–7,122.88)	6.47 (5.68–7.62)	2,299.52 (1,954.31–2,718.03)	4,268.41 (3,607.37–5,059.15)	4.92 (4.25–5.75)
2005	2,771.03 (2,340.65–3,301.39)	4,783.82 (4,083.19–5,645.05)	6.95 (5.89–8.17)	3,314.54 (2,712.82–4,081.45)	5,650.91 (4,725.94–6,933.76)	7.89 (6.47–9.61)	2,237.54 (1,899.38–2,613.22)	3,926.22 (3,352.48–4,564.62)	5.93 (5.15–6.97)
2010	3,087.73 (2,633.3–3,632.49)	4,876.78 (4,219.72–5,710.56)	8.86 (7.6–10.29)	3,825.4 (3,162.95–4,668.03)	5,957.05 (4,991.44–7,260.62)	10.34 (8.59–12.54)	2,386.12 (2,058.06–2,781.41)	3,835.65 (3,350.44–4,445.98)	7.27 (6.38–8.29)
2015	3,424.62 (2,844.54–4,081.97)	4,983.96 (4,199.06–5,871.84)	10.13 (8.75–11.71)	4,386.63 (3,563.06–5,366.39)	6,323.54 (5,182.07–7,684.38)	11.97 (10.07–14.33)	2,534.06 (2,070.69–3,041.36)	3,739.06 (3,087.2–4,430.64)	8.13 (6.74–9.53)
2019	3,631.18 (2,926.9–4,340.92)	4,962.14 (4,021.22–5,854.19)	11.89 (10.14–13.82)	4,668.02 (3,666.2–5,645.85)	6,379.73 (5,049.35–7,676.34)	14.28 (11.95–16.95)	2,686.89 (2,129.38–3,297.54)	3,687.93 (2,940.81–4,488.52)	9.41 (7.65–11.31)

**Table 5:** DALYs per 100,000 and the proportion of DALYs attributable to different CVDs out of total DALYs in 2019.

Causes	Both sex			Male			Female		
	DALYs per 100,000 (95% UI)		% of total DALYs (95% UI)	DALYs per 100,000 (95% UI)		% of total DALYs (95% UI)	DALYs per 100,000 (95% UI)		% of total DALYs (95% UI)
	All age	Age		All age	Age standardized		All age	Age	
		standardized						standardized	
Ischemic heart disease	1,824.07 (1,420.89–2,251.88)	2,490.53 (1,961.78–3,040.38)	5.97 (4.9–7.15)	2,594.11 (1,969.29–3,247.33)	3,543.47 (2,716.66–4,396.01)	7.93 (6.38–9.57)	1,122.76 (805.3–1,469.24)	1,548.03 (1,118.11–2,018.9)	3.93 (2.88–5.08)
Stroke	1,140.06 (897.32–1,422.67)	1,579.32 (1,252.32–1,944.02)	3.74 (3.08–4.57)	1,376.24 (1,053.44–1,750.56)	1,904.58 (1,472.68–2,424.44)	4.21 (3.41–5.32)	924.97 (706.25–1,210.44)	1,284.98 (983.5–1,659.33)	3.24 (2.51–4.21)
Rheumatic heart disease	300.01 (204.08–416.83)	358.56 (240.44–506.56)	0.98 (0.7–1.33)	309.55 (194–533.81)	374.19 (228.49–679.06)	0.95 (0.62–1.58)	291.31 (184.18–462.22)	343.38 (212.74–543.86)	1.02 (0.65–1.58)
Hypertensive heart disease	154.58 (109.49–212.26)	232.62 (166.91–318.96)	0.51 (0.37–0.69)	142.96 (92.71–202.82)	211.18 (139.39–297.93)	0.44 (0.29–0.62)	165.16 (97.73–263.42)	251.44 (149.37–398.5)	0.58 (0.34–0.91)
Other cardiovascular and circulatory diseases	80.79 (58.39–114.6)	104.87 (75.46–149.75)	0.26 (0.2–0.36)	88.82 (60.58–140.37)	116.28 (78.93–185.73)	0.27 (0.2–0.42)	73.47 (54.34–99.26)	94.29 (69.47–130.29)	0.26 (0.19–0.34)
Atrial fibrillation and flutter	61.14 (46.05–79.35)	100.29 (75.42–129.72)	0.2 (0.16–0.25)	65.75 (46.03–88.89)	107.57 (75.13–145.22)	0.2 (0.14–0.26)	56.95 (41.26–74.64)	93.8 (67.34–123.92)	0.2 (0.15–0.25)
Aortic aneurysm	22.61 (14.05–31.44)	31.66 (19.87–43.74)	0.07 (0.05–0.1)	32.15 (18.34–49.16)	44.74 (25.95–67.57)	0.1 (0.06–0.15)	13.92 (9.14–19.52)	19.84 (13.14–27.48)	0.05 (0.03–0.07)
Nonrheumatic valvular heart disease	17.51 (12.72–23.4)	24.48 (17.87–32.63)	0.06 (0.04–0.08)	19.42 (12.36–27)	27.02 (17.12–38.13)	0.06 (0.04–0.08)	15.77 (9.93–23.19)	22.18 (14.07–33.36)	0.06 (0.04–0.08)
Cardiomyopathy and myocarditis	12.38 (8.29–18.33)	15.56 (10.43–22.78)	0.04 (0.03–0.06)	17.54 (11.26–25.87)	21.97 (13.89–32.34)	0.05 (0.04–0.08)	7.68 (4.26–13.74)	9.75 (5.47–17.37)	0.03 (0.02–0.05)
Endocarditis	12.31 (8.72–16.36)	15.2 (10.93–20.18)	0.04 (0.03–0.05)	15.01 (9.73–20.92)	18.57 (11.97–25.97)	0.05 (0.03–0.06)	9.86 (6.15–14.85)	12.18 (7.59–18.12)	0.03 (0.02–0.05)
Peripheral artery Disease	5.72 (3.65–8.43)	9.06 (5.91–13.36)	0.02 (0.01–0.03)	6.47 (3.98–9.38)	10.15 (6.33–14.61)	0.02 (0.01–0.03)	5.05 (3.08–8.06)	8.07 (4.97–12.82)	0.02 (0.01–0.03)

## Data Availability

The data used in the study are publicly available at the official website of the Institute for Health Metrics and Evaluation and can be downloaded from: https://vizhub.healthdata.org/gbd-results/.

## Supplementary Materials

Supplementary Materials Supplementary file 1: data on the age specific mortality rate from CVDs per 100,000 population and the proportion of deaths attributable to CVDs in each age group disaggregated by sex in 2019. Supplementary file 2: data on DALYs attributable to CVDs per 100,000 population and the proportion of DALYs attributable to CVDs in each age group disaggregated by sex in 2019.
