# Supplementary material for: Burden of Cardiovascular Diseases in Nepal from 1990 to 2019: The Global Burden of Disease Study, 2019
Source: Glob Health Epidemiol Genom. 2023 Jun 19;2023:3700094. doi: 10.1155/2023/3700094 (PMC10292936; doi:10.1155/2023/3700094)
Supplement: Supplementary Materials — Supplementary file 1: data on the age specific mortality rate from CVDs per 100,000 population and the proportion of deaths attributable to CVDs in each age group disaggregated by sex in 2019. Supplementary file 2: data on DALYs attributable to CVDs per 100,000 population and the proportion of DALYs attributable to CVDs in each age group disaggregated by sex in 2019. [file 3700094.f1.zip › Supplementary file 1 age specific mortality rate.pdf]

Supplementary material 1: Mortality rate (per 100,000 population) and % of deaths attributable to CVDs

Age	Both sex		Male		Female	
	**Mortality rate (95% UI)**	**% of total deaths (95% UI)**	**Mortality rate (95% UI)**	**% of total deaths (95% UI)**	**Mortality rate (95% UI)**	**% of total deaths (95% UI)**
<1 year	3.08 (1.79-5.08)	0.12 (0.07-0.18)	4.13 (2.17-7.39)	0.15 (0.09-0.25)	1.97 (0.92-3.74)	0.08 (0.04-0.14)
1 to 4	0.41 (0.22-0.72)	0.43 (0.24-0.77)	0.53 (0.26-0.97)	0.56 (0.29-1.01)	0.28 (0.13-0.53)	0.3 (0.14-0.55)
5 to 9	0.42 (0.26-0.63)	0.91 (0.58-1.31)	0.45 (0.26-0.74)	0.88 (0.5-1.42)	0.38 (0.22-0.61)	0.94 (0.56-1.42)
10 to 14	0.8 (0.5-1.24)	2.02 (1.28-3.03)	0.95 (0.48-1.61)	2.06 (1.09-3.54)	0.65 (0.35-1.09)	1.96 (1.08-3.19)
15 to 19	4.46 (2.92-6.54)	5.53 (3.79-7.86)	6.48 (3.85-10.23)	6.15 (3.87-9.65)	2.5 (1.34-3.99)	4.41 (2.61-6.88)
20 to 24	7.55 (4.81-10.98)	7.15 (5.05-10.12)	11.68 (6.63-18.52)	8.06 (5.05-12.48)	3.94 (2.28-5.87)	5.53 (3.41-8.14)
25 to 29	9.35 (5.69-13.46)	8.3 (5.57-11.5)	13.2 (7.39-21.11)	9.19 (5.42-14.21)	6.3 (3.42-9.57)	7.14 (4.17-10.51)
30 to 34	15.94 (8.55-23.62)	11.47 (6.58-15.31)	21.51 (9.07-34.48)	12.8 (5.72-19.46)	11.77 (6.17-18.52)	10.03 (5.68-14.46)
35 to 39	27.94 (15.31-39.5)	14.28 (8.22-18.6)	36.36 (12.4-55.97)	15.98 (5.77-23.02)	21.61 (12.96-33.22)	12.58 (7.8-17.88)
40 to 44	58.32 (38.87-82.23)	19.66 (14.05-24.41)	73.67 (43.26-107.66)	21.6 (13.01-28.56)	46.08 (29.69-66.67)	17.65 (12.27-23.43)
45 to 49	107.91 (75.08-145.5)	23.31 (17.99-28.39)	135.82 (86.26-192.85)	25.54 (18.24-33.01)	83.81 (55.1-118.12)	20.77 (15.03-27.28)
50 to 54	197.83 (142.74-261.17)	26.64 (22.26-31.34)	266.79 (188.49-361.34)	30.46 (23.79-38.19)	133.94 (87.2-190.45)	21.64 (15.5-28.12)
55 to 59	337.22 (246.71-435.76)	29.06 (24.67-33.66)	455.04 (327.45-597.44)	32.39 (25.91-38.54)	225.7 (153.69-316.25)	24.3 (17.67-31.28)
60 to 64	553.58 (405.1-706.51)	29.83 (25.22-35.17)	753.42 (533.17-973.27)	33.06 (26.25-40.29)	369.03 (258.02-500.54)	25.19 (18.95-31.89)
65 to 69	879.73 (656.13-1098.98)	30.44 (26.03-35.39)	1188.42 (854.39-1516.94)	33.81 (27.82-40.08)	606.43 (423.09-808.8)	25.95 (19.18-32.87)
70 to 74	1393.55 (1067.93-1724.36)	30.04 (25.02-35.31)	1778.76 (1283.64-2290.41)	33.06 (26.5-40.34)	1028.11 (710.9-1354.5)	26.12 (18.69-33.87)
75 to 79	2227.02 (1756.21-2709.09)	30.41 (25.57-35.78)	2671.6 (2013-3363.06)	32.81 (26.39-39.92)	1805.85 (1274.3-2350.23)	27.59 (19.94-35.17)
80 plus	4081.43 (3343.6-4794.57)	27.49 (23.73-31.36)	4958.56 (4013.36-5924.39)	31.56 (27.05-37.21)	3323.11 (2508.31-4258.97)	23.56 (18.23-29.3)
